# Is There a Future for CAR-T Therapy in Acute Myeloid Leukemia?

**DOI:** 10.3390/cancers18010107

**Published:** 2025-12-29

**Authors:** Caterina Alati, Martina Pitea, Matteo Molica, Marco Rossi, Maria Eugenia Alvaro, Gaetana Porto, Erica Bilardi, Giovanna Utano, Giorgia Policastro, Maria Caterina Micò, Violetta Marafioti, Massimo Martino

**Affiliations:** 1 Hematology and Stem Cell Transplantation and Cellular Therapies Unit (CTMO), Department of Hemato-Oncology and Radiotherapy, Grande Ospedale Metropolitano “Bianchi-Melacrino-Morelli”, 89133 Reggio Calabria, Italyeribil95@gmail.com (E.B.); mariacaterina.mico@ospedalerc.it (M.C.M.); dr.massimomartino@gmail.com (M.M.); 2 Department of Hematology-Oncology, Azienda Universitaria Ospedaliera Renato Dulbecco, 88100 Catanzaro, Italy; molica@bce.uniroma1.it (M.M.); rossim@unicz.it (M.R.); 3 Division of Hematology, Department of Human Pathology in Adulthood and Childhood “Gaetano Barresi”, University of Messina, via Consolare Valeria, 98125 Messina, Italy; m.eugenia983@gmail.com

**Keywords:** acute myeloid leukemia, AML, CAR-T therapy, treatment, tumor microenvironment, leukemia-specific antigen

## Abstract

Acute myeloid leukemia (AML) is an aggressive cancer with rapid progression and a high relapse rate, highlighting the urgent need for effective treatments. While recent advances in drug therapies and combination regimens have improved outcomes, relapsed and refractory (R/R) AML still shows low response rates, poor prognosis, and limited survival. The lack of effective immunotherapies further complicates the management of R/R AML. The bone marrow tumor microenvironment (TME) poses a significant barrier, requiring multifaceted, combined therapeutic strategies for clinical success. This TME creates an immunosuppressive and metabolically challenging environment that limits the expansion, persistence, cytotoxicity, and survival of chimeric antigen receptor (CAR) T cells. Unlike CD19 in B-cell acute lymphoblastic leukemia (B-ALL), AML lacks a truly leukemia-specific antigen. Although clinical trials are ongoing, no CAR-T therapies have received FDA approval for AML. This paper explores the reasons behind these ongoing challenges.

## Simple Summary

Acute myeloid leukemia (AML) is a dismal disease with a poor prognosis, particularly in the relapsed and refractory (R/R) setting, and there is an urgent need for new treatments. CAR-T therapy has produced remarkable clinical results in treating other hematological diseases. Therefore, it has the potential to achieve similar outcomes in R/R AML. However, its effectiveness is hindered by the difficulty of identifying appropriate target antigens and of creating effective receptors to recognize and safely eradicate AML cells.

## 1. Introduction

Acute myeloid leukemia (AML) is a hematologic malignancy characterized by the clonal expansion of myeloid blasts in the bone marrow (BM), peripheral blood, or other tissues. AML represents the most common form of acute leukemia (AL) in adults and is responsible for the highest rate of AL-related mortality worldwide [1]. The median age at diagnosis is 68 years, with approximately 60% of patients diagnosed at 65 years of age or older and 33% at 75 years of age or older [2]. While multiagent induction chemotherapy can achieve complete remission (CR), allogeneic stem cell transplantation (allo-SCT) remains the only established curative treatment [3]. Despite recent advances in therapeutic strategies, prognosis remains poor, particularly in older patient populations [4].

Chimeric antigen receptor T (CAR-T) cell therapies represent a promising and innovative approach for treating hematologic malignancies. These therapies are classified as Advanced Therapy Medicinal Products (ATMPs) under European Union regulations, specifically as cell-based gene therapy products [5]. In the United States, CAR-T cells are regulated by the Food and Drug Administration’s Center for Biologics Evaluation and Research as biological products [6]. This regulatory framework defines them as medicinal products consisting of autologous or allogeneic cells that have been genetically modified to express chimeric antigen receptors. The ATMP designation imposes specific regulatory requirements across development, manufacturing, and clinical application, including stringent Good Manufacturing Practice (GMP) standards, comprehensive quality control, and extensive safety surveillance procedures that distinguish these therapies from conventional treatments [7,8].

Given the limited curative options for AML, particularly in elderly patients and those with relapsed (R) or refractory (R) disease, and considering the distinct regulatory and clinical challenges of CAR-T cell therapy as an ATMP, a comprehensive review of recent advances, target antigens, clinical outcomes, and implementation strategies for CAR-T therapy in AML is necessary to inform future research and clinical practice.

## 2. Induction Therapy

The initial treatment strategy for AML depends on several factors, including patient performance status and functional status, which determine tolerance to standard induction therapy, prior exposure to radiotherapy or chemotherapy, and the presence of specific cytogenetic and molecular markers [3,9,10,11]. Cytarabine (Ara-C) combined with anthracyclines remains the standard chemotherapy regimen for patients eligible for intensive therapy. Alternative regimens include idarubicin, fludarabine, cytarabine, and mitoxantrone-based cytarabine combinations. Additionally, patients aged 18 to 75 years with FLT3-ITD mutations who are receiving standard 7 + 3 induction therapy should be treated with quizartinib, according to the QUANTUM study [12]. When acute promyelocytic leukemia (APL) is suspected, treatment with all-trans retinoic acid (ATRA) is recommended [3].

The standard regimen for older patients, typically those aged 70 years or above, as well as for fit individuals, consists of a hypomethylating agent, such as azacitidine or decitabine, combined with venetoclax, a Bcl-2 inhibitor and BH3 mimetic [13,14,15,16]. Additionally, the oral formulation of decitabine with cedazuridine broadens therapeutic options for AML and enhances the convenience of administration [17].

In recent years, the Food and Drug Administration (FDA) and the European Medicines Agency (EMA) have approved multiple drugs and drug combinations for the treatment of AML. These regulatory approvals offer additional therapeutic options for patients with varying disease characteristics (see Table 1) [18,19,20,21,22,23,24,25].

## 3. Consolidation Therapy

Even after achieving a CR to optimal induction therapy, minimal residual disease (MRD) often remains. Consolidation therapy is required to eliminate residual disease and reduce relapse risk [3]. For patients treated with 7 + 3 induction therapy, consolidation therapy begins with a high-dose cytarabine regimen. Those who received FLAG during induction should continue with additional cycles of the same regimen during consolidation.

All eligible patients with intermediate- or high-risk disease should be offered allo-SCT, regardless of the treatment regimen [3]. AML is the most common indication for allo-SCT [31,32,33]. The decision to proceed with allo-SCT in first remission depends on the risk–benefit ratio, which considers nonrelapse mortality and relapse reduction. This assessment is based on cytogenetic and molecular features at diagnosis, response to initial therapy, and patient, donor, and transplant factors [3,33].

## 4. Maintenance Therapy

Maintenance therapy refers to prolonged treatment given after a patient achieves remission to prevent relapse. Unlike ALL, where maintenance therapy is standard [34], its role in AML is more limited and context-specific [3,35].

## 5. How to Treat Relapsed or Refractory (R/R) AML

Several agents are available for patients with R/R AML [26,27,28]. FLT3 inhibitors, such as gilteritinib [29] and quizartinib [30], have shown higher CR rates than salvage chemotherapy in this population. Patients with IDH1 mutations, older patients, and those unfit for induction therapy should be offered ivosidenib [36] or olutasidenib [37]. Patients with IDH2 mutations may be offered enasidenib [38]. Recently, ziftomenib and revumenib received FDA approval for R/R AML with a susceptible NPM1 mutation or KMT2A gene rearrangement, marking significant progress for these genetic subtypes [39].

Despite significant advances, R/R AML remains associated with poor prognosis, lower response rates, and shorter survival compared to newly diagnosed AML [40]. Patients who relapse after initial treatment or are refractory to standard therapies typically have a median overall survival (OS) of six to twelve months. Poor prognosis is linked to advanced age, unfavorable cytogenetics, prior allo-SCT, and failure to achieve another CR [41].

## 6. CAR-T in Relapsed or Refractory AML

R/R AML remains challenging because effective immunotherapies are lacking [42,43,44]. Although clinical trials are evaluating CAR-T therapy for AML [45,46,47,48,49,50,51,52,53,54,55,56], none have received FDA approval.

Lin et al. evaluated CD33 CAR T cells in 12 patients with relapse after allo-SCT [57]. They reported a 41.67% CR rate, with no grade 3–4 cytokine release syndrome (CRS) or immune effector cell-associated neurotoxicity syndrome (ICANS).

Naik et al. investigated CD123 CAR T cells manufactured with dasatinib (CD123-CAR.dasa T cells) in six patients, including five with AML and one with ALL. While CD123-CAR.dasa T cells demonstrated effective expansion, they did not show improved outcomes compared to conventional CD123 CAR T cells. Furthermore, all six patients experienced grade 2 or higher cytokine release syndrome (CRS), raising significant concerns regarding toxicity [58].

Zhao et al. reported outcomes of CLL1 CAR T cell therapy in 47 patients with rR/R AML, including 20 with extramedullary infiltration. Patients with and without extramedullary infiltration exhibited similar OS and leukemia-free survival (LFS) rates, as well as identical complication rates [59]. Additionally, three patients received modified CLL1-CARs incorporating a KDEL tag and anti-CD3 (Tis-CART371). Two of these patients achieved a CR, but ultimately, all patients died [60]. Infection remains a significant concern following CAR T-cell therapy. The authors reported a 56.9% cumulative rate (95% CI 50.4–61.3%) of bacterial, fungal, and viral infections within 28 days [61].

A phase I trial of the novel CD371-SAVVYz-IL18 CAR T cell achieved CR with MRD negativity in three of five patients. This CAR T cell incorporates a modified CD28 costimulatory domain to reduce T cell exhaustion and constitutive IL-18 secretion, thereby enhancing cytotoxicity. However, high-grade CRS and ICANS were observed [62].

A trial of CD19 CAR T cells in ten patients with t(8;21) AML demonstrated a favorable safety profile, with no severe nonhematologic toxicities, and a 100% response rate. Sixty percent of patients achieved MRD-negative complete remission. The 12-month OS and LFS were 45.0% and 46.7%, respectively [63].

## 7. Open Issues and Solutions

The success of CAR-T therapy in AML has been limited by clonal heterogeneity [45,64,65], a highly immunosuppressive bone marrow microenvironment [51], and a lack of tumor-specific target antigens [66,67].

The bone marrow tumor microenvironment in AML significantly impairs T-cell function [68]. Myeloid-derived suppressor cells (MDSCs) and regulatory T cells (Tregs) contribute to this immune suppression, affecting both endogenous and adoptively transferred T cells [69]. AML cells often reduce or lose major histocompatibility complex (MHC) expression, which is essential for antigen presentation to T cells. This impairs immune clearance of leukemia cells, allowing cancer cells to evade detection and potentially limiting the effectiveness of engineered T-cell therapies [70].

To improve T-cell persistence, T cells can be engineered to resist the immunosuppressive microenvironment [71,72]. The AML microenvironment contains high levels of immunosuppressive cytokines (such as TGF-β), regulatory cells (Tregs and MDSCs), and immune checkpoints (such as the PD-1/PD-L1 axis), all of which can inactivate T cells [42]. Strategies to overcome these barriers include engineering T cells to secrete activating cytokines like IL-12 [73] or IL-18 [74] at the tumor site, modifying T cells to express dominant-negative receptors that block immunosuppressive signals [75], and combining T cell therapy with systemic immune checkpoint inhibitors (ICIs) [76] or hypomethylating agents (HMAs), such as azacitidine and decitabine [77]. ICIs can enhance the antitumor activity of CAR-T cells by reducing the secretion of inhibitory cytokines and partially reversing the suppressive effects of the tumor microenvironment [78]. They also activate tumor-infiltrating lymphocytes (TILs) and improve their antitumor function [79].

Combining CAR-T cell therapy with HMAs is a promising approach, as HMAs can increase the expression of target antigens (such as CD70 and CD123) on AML cells by reducing promoter methylation [80]. This makes leukemia cells more susceptible to CAR-T cell targeting. Pre-treatment with HMAs has also been shown to reduce T cell exhaustion after infusion, potentially improving CAR-T cell persistence and antitumor activity [81]. The heterogeneity in target antigens in AML poses a significant challenge to the effectiveness of CAR-T cell therapy. Antigens such as CD33, CD123, and CLL-1, which are commonly targeted in AML, are not uniformly expressed on all leukemic cells [66,67]. Moreover, many potential AML-associated antigens, such as CD33 and CD123, are also expressed on healthy hematopoietic stem and progenitor cells, increasing the risk of on-target, off-tumor toxicity [82].

The following AML-specific targets have been identified as promising and are currently under investigation (Table 2): CD33 [83], CD123 [84], CLL-1 [85], NKG2D ligand [86], CD43 [87], CD96 [88], interleukin-3 receptor alpha chain [89], TIM-3 [90], CD38 [91], CD157 [92], CD44v6 [93], and CD47 [94], among others [95]. CD64-directed CAR T cell therapy has been shown to effectively treat Ven/Aza-resistant monocytic AML [96]. Mutations such as FLT3-ITD, NPM1, and DNMT3A contribute to AML pathogenesis and influence responses to CAR-based therapies, emphasizing the need for molecularly guided treatment strategies [97]. Finally, Knorr et al. described a therapeutic antibody targeting an AML-associated antigen, U5 snRNP200, which we have demonstrated to be limited to malignant cells and not expressed on normal HSPCs [98].

Single-target CAR-T therapy may not eliminate all leukemia cells because AML blasts express different antigens, increasing the risk of relapse [99]. Targeting multiple antigens with armored or multispecific CAR-T cells can improve outcomes [100]. Alternatively, adapter CARs use a soluble molecule to link CAR T cells to target antigens, offering greater flexibility and control [101].

T-cell persistence—their ability to survive, multiply, and remain active after infusion—is essential for long-term remission. Strategies include optimizing manufacturing and laboratory growth conditions to infuse less differentiated, more resilient cells that persist longer [102]. Genetic modifications to enhance T-cell metabolism and resistance to exhaustion are also used [103].

“Off-the-shelf” CAR-T cell therapy uses T cells from healthy donors, manufactured in large batches [104,105]. This approach addresses limitations of traditional CAR-T therapy, including high costs, long manufacturing times, and poor-quality patient cells. The key challenges are preventing graft-versus-host disease (GVHD) and host-versus-graft rejection [106].

## 8. Chimeric Antigen Receptor Natural Killer (CAR-NK)

CAR-NK cell therapy is a promising alternative to CAR-T cells. Natural killer (NK) cells are innate immune effectors that can directly kill tumor cells without prior antigen sensitization [107]. CAR-NK cells can be sourced from peripheral blood, cord blood, or NK cell lines, such as NK-92 [108]. They offer a more favorable safety profile, with a significantly reduced risk of CRS and ICANS due to more regulated cytokine secretion [109]. CAR-NK cells also provide manufacturing advantages as an “off-the-shelf” product and can be produced from universal donors [110].

Preclinical and early clinical data show promising antitumor efficacy of CAR-NK cells targeting CD19, BCMA, and other tumor-associated antigens, with a lower incidence of GVHD [111]. However, as CAR-NK trials are recent, there is limited literature on the impact of AML mutations on CAR-NK cell therapy.

## 9. Expert Opinion

Developing CAR-T therapy for AML is among the most complex challenges in current immunotherapy. While CAR-T therapies have transformed B-cell malignancy treatment with CR rates of 60–90%, results in AML remain modest, with response rates typically below 50% and limited durability [112,113]. This gap is due to key biological differences between AML and lymphoid malignancies, including antigen heterogeneity, lack of AML-specific targets, an immunosuppressive tumor microenvironment, and unique manufacturing challenges (Table 3).

A key challenge is that the bone marrow TME in AML creates a complex, immunosuppressive, and metabolically hostile environment that impairs CAR T-cell expansion, persistence, cytotoxicity, and survival. Conventional CAR T-cell constructs cannot overcome the combined barriers of immunosuppression, metabolic stress, and physical sequestration in the AML bone marrow. This limitation explains the high response rate of about 90% in B-ALL, compared to only 20–40% in early AML trials (Table 4). The limited efficacy of CAR T-cell therapy in AML results from a mismatch between CAR T-cell biology, which is optimized for circulating B-cell malignancies, and the unique immunometabolic conditions of the AML bone marrow. Success will require comprehensive re-engineering of CAR T cells for the bone marrow environment, along with targeted TME conditioning strategies.

Unlike B-ALL, where CD19 is a reliable target, AML lacks a leukemia-specific antigen, and CAR-T cells are prone to exhaustion in myeloid leukemia. The MDSC and Treg axis is the main cellular suppressive mechanism. Depleting MDSCs or suppressing Tregs should occur before CAR T-cell infusion. In this context, checkpoint blockade should be used prophylactically, not as a rescue strategy, and combining it with hypomethylating agents has synergistic effects. Further interventions are needed. A key driver of AML relapse is the protection of the leukemic stem cell (LSC) niche. Relapse can occur even after achieving MRD negativity because LSCs persist in CXCR4/CXCL12-mediated endosteal niches that CAR T cells cannot access [114].

The “cytokine sink” phenomenon in AML remains poorly understood. AML blasts and stromal cells consume interleukins such as IL-2, IL-7, and IL-15, which are essential for CAR T-cell expansion, while secreting antagonistic cytokines, such as IL-10 and TGF-β. This underscores the need for localized cytokine delivery. Production of autologous CAR-T cells from AML patients is often compromised, as their T cells frequently exhibit intrinsic dysfunction due to prior chemotherapy, disease-related immunosuppression, and aging [115]. Current CAR-T constructs in AML reach peak activity too early and then quickly exhaust. Pharmacokinetic analyses show that AML CAR T cells peak between days 7 and 10, compared to days 14 and 21 in ALL, and decline by day 21 [116]. This short window may be insufficient to eliminate slow-cycling leukemic stem cells. The therapeutic window for CAR-T efficacy closes rapidly after infusion [117]. Rapid and profound responses are critical given AML’s doubling time of 10 to 30 days. Furthermore, the 2-to-4-week manufacturing period for autologous products may allow for disease progression during production.

Manufacturing and GMP compliance pose significant challenges for CAR-T cell therapy in AML. The process faces regulatory hurdles at several stages of development. Autologous production requires individualized, multi-step procedures with strict controls over transport, storage, and processing, which can lead to variability in product quality, including cell viability, potency, and identity [5]. Each stage, from leukapheresis and T-cell isolation to genetic modification, expansion, final formulation, and cryopreservation, must meet GMP standards and undergo thorough validation [118]. Production is further limited by costs, raw material availability, equipment, and environmental controls, while culture media and reagents remain unstandardized. The complexity of preparation and the need for advanced genetic modification technologies create substantial financial barriers, with treatment costs exceeding USD 500,000 per patient [119]. In AML, further complications include the risk of blast contamination in autologous products and reduced T-cell fitness due to prior intensive chemotherapy [120].

Regulatory agencies require comprehensive preclinical safety and efficacy data before first-in-human trials. However, preclinical models often do not reflect the full spectrum of human toxicities, especially those specific to AML, such as CRS and ICANS. The autologous nature of AML CAR-T products adds regulatory complexity. Site training, deviation protocols, and shipping validation have typically required Risk Evaluation and Mitigation Strategy (REMS) programs. The FDA has recently removed REMS requirements for certain approved autologous CAR-T therapies [118]. Despite this, vein-to-vein times of 3 to 6 weeks still cause life-threatening delays for patients with aggressive AML [64].

Most CAR-T clinical trials are small, single-center studies with limited long-term follow-up. Regulatory agencies now require standardized trial designs to validate safety and efficacy. Due to the long-term persistence of CAR-T cells and the potential risk of insertional mutagenesis, developers must conduct extended follow-up, often up to 15 years post-treatment, to monitor for delayed adverse events and immunogenicity [118]. These requirements may be reduced for products with low persistence, but such changes require strong justification and early agreement with the FDA. Only 35% of CAR-T trials progress beyond Phase 2, reflecting significant regulatory and scientific barriers. The lack of FDA-approved CAR-T products for AML further underscores these challenges [121].

CAR-T therapy requires specialized clinical settings with intensive care capabilities to manage life-threatening toxicities. Healthcare professionals must be trained in both CAR-T administration and toxicity management [122]. The need for ongoing intensive care, specialized pharmacy support, and long-term monitoring limits the number of centers that can safely provide CAR-T therapy.

Allogeneic “off-the-shelf” CAR-T products can address several challenges of autologous manufacturing. They reduce production time by over six weeks, provide superior cell sources, enable easier scaling and standardization under GMP conditions, and allow storage of multiple therapeutic doses. However, these products raise new regulatory concerns, including the risks of graft-versus-host disease (GvHD) and allo-rejection. Advanced gene-editing strategies, such as CRISPR/Cas9, are required to eliminate T-cell receptor (TCR) and human leukocyte antigen (HLA) molecules [123].

The utility and limitations of preclinical studies in AML remain a significant concern. Animal models often fail to predict the clinical behavior of CAR-T cells, and cell therapy applications differ from regulatory expectations more than other biologic agents, especially in toxicology and mechanisms of action [124]. While xenograft models are the most common preclinical platform, they have notable limitations for AML CAR-T research. These models provide limited guidance for selecting CAR-T cell doses for first-in-human trials, as preclinical doses are typically much higher than clinically safe levels. Immunocompromised mice require higher CAR-T doses to show antitumor effects [125]. Differences in antigen specificity, density, and expression between species further complicate translation. Xenoreactivity from the original T-cell receptor repertoire also limits the duration of experiments. Furthermore, xenograft models do not replicate the immunosuppressive AML microenvironment, including MDSCs, regulatory T cells, and inhibitory cytokines that significantly affect CAR-T function in patients [126].

AML presents unique in vitro challenges. Many AML cell antigens are shared with healthy hematopoietic cells, and their variable expression complicates tumor targeting. Recent studies show that myeloid-supporting cytokines released during cell therapy promote AML blast survival via kinase signaling pathways, leading to CAR-T cell exhaustion. This resistance mechanism differs from those in B-cell malignancies [127] and cannot be effectively modeled in standard xenograft systems. Additionally, AML creates a hostile microenvironment through MDSCs, regulatory T cells, increased checkpoint molecules, and other immunosuppressive factors, which are not adequately represented in immunodeficient mouse models.

Major histocompatibility complex (MHC) restriction remains a significant challenge. Traditional T-cell receptor (TCR)-based therapies depend on antigen presentation via MHC molecules, which limits their use to certain human leukocyte antigen (HLA) types and makes them vulnerable to immune escape through MHC downregulation [128]. CAR technology overcomes this by using antibody-derived binding domains, allowing T-cell recognition and activation without MHC presentation, and enabling targeting of any antigen for which an available antibody is available. However, in AML, MHC-independence alone does not address all biological barriers. To further improve CAR-T efficacy, advanced strategies have been developed, including the use of γδ T cells, which represent a paradigm shift in MHC-independent cellular therapy [129]. γδ T cells can target extracellular domains and induce cytotoxicity and cytokine production without MHC presentation; they make up a small subset of peripheral blood cytotoxic T cells [130]. Because γδ T cells lack an MHC-dependent TCR, they have not been linked to GvHD and are promising for allogeneic “off-the-shelf” CAR-T therapies, addressing manufacturing and accessibility issues and removing the need for HLA matching. NK cells are another MHC-independent platform. Both NK and γδ T cells bridge innate and adaptive immunity and can target tumors without MHC, making them suitable for allogeneic cell therapies. NK cells recognize targets through a balance of inhibitory and activating receptors. Tumor cells often downregulate MHC-I, enabling “missing-self” recognition, and upregulate stress ligands that activate NK cell receptors [131]. CAR-NK cells offer several advantages, including a lower risk of cytokine release syndrome, shorter lifespan, fewer long-term safety concerns, and suitability for off-the-shelf products. Multiple cell sources are being studied, such as peripheral blood NK cells, umbilical cord blood NK cells, and induced pluripotent stem cell (iPSC)-derived NK cells [132].

The optimal treatment strategy for newly diagnosed AML patients remains uncertain. Intensive chemotherapy achieves initial complete remission rates of 50–70%, but five-year overall survival for adults is below 30%, with relapse as the primary cause of treatment failure [133,134]. This high relapse risk supports considering CAR-T therapy, though the ideal timing and cellular source require further evaluation. In autologous CAR-T manufacturing, patient T-cell quality is critical. Disease factors and prior therapies can impair T-cell fitness, and autologous approaches face additional challenges, including manufacturing delays, dependence on T-cell quality, and risk of blast contamination [135]. Although autologous CAR-T manufacturing is feasible in AML, as demonstrated by a 90.4% production success rate, treatment was associated with high rates of cytokine release syndrome and limited clinical benefit. Early T-cell collection with cryopreservation enables flexible CAR-T infusion timing based on disease status and minimal residual disease (MRD) monitoring [136]. Recent data support using CAR-T therapy as a bridge to allo-SCT for sustained disease control. CD7 CAR-T therapy induced remission in most AML patients, primarily those who relapsed after prior allo-SCT, but durable responses were observed only in patients who subsequently underwent allo-SCT [137].

Cost is a significant barrier to adopting CAR-T therapy in AML and other indications [138,139,140]. Approved CAR-T products range from USD 373,000 to USD 475,000. Patients must remain within two hours of the treatment center for at least four weeks after therapy, leading to substantial out-of-pocket expenses for accommodation, transportation, and lost income for both patients and caregivers. Healthcare institutions also face costs for hiring specialized staff, obtaining REMS certification, and educating patients and caregivers. The highest monthly expenses occur during CAR-T administration, with further costs for managing complications.

No CAR-T therapy has received FDA approval for AML, so all treatments remain investigational. Although cost-effectiveness analyses are available for approved CAR-T products in ALL and lymphoma [141], no published studies are available for AML due to its investigational status. Advancements in manufacturing, research, long-term follow-up, comparative effectiveness studies, and head-to-head clinical trials are needed to reduce uncertainty in economic assessments [140,142,143]. Real-world evidence from large, prospective registries is also necessary to better estimate long-term effectiveness and adverse events, support payment model innovation, and improve accessibility.

Table 5 summarizes potential solutions to improve CAR-T therapy in AML.

## 10. Conclusions

AML has a poor prognosis, especially in relapsed or refractory cases, highlighting the urgent need for new therapies. CAR-T therapy has shown significant success in other hematological malignancies and may offer similar benefits in R/R AML. However, its effectiveness is limited by challenges in identifying suitable target antigens and developing receptors that can safely and efficiently eliminate AML cells. New CAR-T strategies targeting alternative antigens and employing innovative CAR designs are approaching clinical evaluation. Advances such as multiantigen targeting, logic gating, and novel cell engineering can enhance T-cell specificity and sensitivity for AML. To improve outcomes, CAR strategies must address the complex biology of AML through a multifaceted approach. No single modification will overcome all barriers. Optimal AML CAR T cells will likely require logic-gated antigen recognition, checkpoint resistance, metabolic armoring, cytokine autonomy, enhanced trafficking, and niche disruption. The complexity of these modifications is reaching the limits of current vector packaging, emphasizing the need for new delivery platforms.

## Figures and Tables

**Table 1:** Recently approved pharmacological agents for the treatment of acute myeloid leukemia (AML).

Name and References	Characteristics	Indications
CPX-351 [13,15]	Dual-drug liposomal formulation that encapsulates cytarabine/daunorubicin in a 5:1 fixed molar ratio	Adults with newly diagnosed therapy-related AML or AML with myelodysplastic-related changes.
Gemtuzumab-Ozogamicin [16]	Humanized anti-CD33 IgG4 antibody chemically linked to a calicheamicin-based cytotoxic warhead.	In combination with daunorubicin and cytarabine for the treatment of patients aged 15 years and older with de novo, previously untreated CD33-positive AML, except acute promyelocytic leukemia.
Azacitidine [9,11,13]	DNA methylation inhibition. Azacytidine, once incorporated into DNA, irreversibly binds to DNA methyltransferases.	Adult patients who are not eligible for ALLO-SCT with the following: MDS at intermediate 2 and high risk according to the IPSS. CMML with 10–29% bone marrow blasts without myeloproliferative disorder. AML with 20–30% blasts and multilineage dysplasia, according to the WHO classification. AML with >30% bone marrow blasts according to the WHO classification.
Dacitabine [14]	Synthetic nucleoside analogue of cytidine. Once inside the cell, it is incorporated into DNA during replication. When DNA methyltransferases attempt to methylate DNA containing decitabine, they become irreversibly bound to it.	Adult patients with newly diagnosed AML “de novo” or secondary, according to the WHO classification, who are not eligible for standard induction chemotherapy.
Midostaurin [18]	Protein kinase inhibitor. It works by blocking the action of specific proteins called kinases, which play a key role in the growth and division of cancer cells. By inhibiting these kinases, midostaurin helps to slow down or stop the proliferation of cancer cells.	In combination with standard induction chemotherapy with daunorubicin and cytarabine and consolidation with high-dose cytarabine, followed, for patients in complete response, by maintenance therapy as a single agent for adult patients with newly diagnosed AML with FLT3 mutation as monotherapy for the treatment of adult patients with aggressive systemic mastocytosis, systemic mastocytosis with associated hematological neoplasm, or mast cell leukemia.
Gilteritinib [17]	Tyrosine kinase inhibitor. Gilteritinib binds to the adenosine triphosphate (ATP)-binding site of the FLT3 receptor, which is often mutated (e.g., FLT3-ITD and FLT3-TKD mutations) and constitutively active in acute AML cells. Competitive inhibition of this site prevents the receptor from autophosphorylating and activating downstream signaling cascades.	Treatment of adult patients with relapsed or refractory AML that has an FLT3 mutation.
Quizartinib [8,19,26]	Oral and potent FLT3 inhibitor. It is the first drug developed specifically targeting FLT3, as other agents with FLT3 inhibition activities were investigated with other targets in mind. Additionally, quizartinib also demonstrates inhibitory activity toward FLT3 with ITD, although with a 10-fold lower affinity compared to wild-type FLT3.	In combination with standard induction chemotherapy based on cytarabine and anthracycline and standard consolidation chemotherapy based on cytarabine, followed by Quizartinibas maintenance monotherapy, for adult patients with newly diagnosed FLT3-ITD-positiveAML.
Venetoclax [9,10,11,12]	Potent selective inhibitor of BCL-2, an anti-apoptotic protein. Venetoclax binds directly to the BH3-binding domain of BCL-2, preventing the binding of pro-apoptotic proteins (such as BIM) that contain BH3 motifs, resulting in mitochondrial outer membrane permeabilization (MOMP), caspase activation, and programmed cell death.	In combination with a hypomethylating agent, it is indicated for the treatment of adult patients with newly diagnosed AML who are not eligible for intensive chemotherapy.
Ivosidenib [25,27]	First-in-class IDH1 inhibitor. IDH1 is an enzyme that is often mutated and overexpressed in some cancers, leading to aberrant cell growth and proliferation. Ivosidenib inhibits mutated IDH1, blocking its enzymatic activity and preventing the further differentiation of cancer cells.	Newly diagnosed AML in older adults in combination with azacitidine or as monotherapy, and relapsed or refractory myelodysplastic syndromes in adults. The drug is only effective in patients with a susceptible IDH1 mutation.
Olutasidenib [20,28]	Small-molecule inhibitor that works by selectively targeting the mutated IDH1 enzyme, preventing it from producing the oncometabolite 2-hydroxyglutarate.	Adult patients with R/R AML who have a susceptible IDH1 mutation.
Enasidenib [21,29]	It works by selectively inhibiting the mutant form of the IDH2 enzyme. This inhibition decreases the levels of the oncometabolite 2-hydroxyglutarate (2-HG), which, in turn, relieves the block on normal myeloid cell differentiation and promotes the maturation of leukemic cells into more functional white blood cells.	Adult patients with R/R AML who have an IDH2 mutation.
Menin inhibitors [30]	Menin inhibitors work by disrupting the interaction between the menin protein and the KMT2A (or MLL) complex, which is crucial for the survival of certain AML cell types. This disruption blocks the expression of genes such as HOX and MEIS1, leading to the differentiation, proliferation arrest, and apoptosis of cancer cells.	R/R AML, specifically those with either a KMT2A gene rearrangement or an NPM1 mutation. They are approved for adults and children aged one year and older.

Legend: AML, acute myeloid leukemia; WHO, World Health Organization; MDS, myelodysplastic syndrome; IPSS, International Prognostic Scoring System; CMML, chronic myelomonocytic leukemia; FLT3, fms-like tyrosine kinase 3; ITD, internal tandem duplication; BCL-2, B-cell lymphoma 2; IDH1, isocitrate dehydrogenase 1; IDH2, isocitrate dehydrogenase 2.

**Table 2:** Emerging CAR-T cell therapy targets in acute myeloid leukemia (AML).

Target (Reference)	Expression
CD33 [83]	Expressed in more than 90% of leukemic blasts. Expressed in normal progenitor cells, myeloid cells, monocytes, and tissue-resident macrophages.
CD123 [84]	Cell-surface markers are overexpressed on many AML cells. This overexpression is particularly noted in AML subtypes with NPM1 and/or FLT3 mutations, which may benefit from CD123-targeted treatments.
CLL-1 [85]	Highly expressed on AML blasts and demonstrates stable expression throughout disease progression. CLL-1’s consistent presence makes it an ideal candidate for monitoring minimal residual disease (MRD), which is a critical indicator for predicting relapse.
NKG2D ligand [86]	Proteins expressed on the surface of AML cells that are recognized by the NKG2D receptor on immune cells, like natural killer (NK) and T cells, which can then trigger an immune response to kill cancer cells. While some AML cells express a variety of NKG2D-L, others, particularly leukemia stem cells, often evade immune detection by downregulating or expressing low levels of these ligands.
CD43 [87]	A protein that is being studied as a potential new therapeutic target for AML, specifically a form called CD43s, which is present in leukemia cells but not in healthy cells.
CD96 [88]	Marker of leukemic stem cells (LSCs) in AML cells. Its expression is associated with tumor cell proliferation and dysfunction of natural killer (NK) cells that attack tumor cells. Therapies targeting CD96 could help eliminate residual LSCs and improve treatment outcomes, particularly in stem cell transplants.
interleukin-3 receptor alpha chain [89]	Also called CD123, it is a cell-surface protein that is a promising therapeutic target in AML because it is overexpressed on AML stem cells but not on normal stem cells. Targeting CD123 enables the selective destruction of leukemia cells with minimal impact on healthy blood cells. High CD123 expression in AML is associated with a worse prognosis and higher blast counts at diagnosis.
TIM-3 [90]	T-cell immunoglobulin mucin-3 (TIM-3) is expressed on LSCs in most types of primary AML, except for acute promyelocytic leukemia. TIM-3 is not expressed in normal hematopoietic stem cells (HSCs). Targeting TIM-3 with an anti-TIM-3 cytotoxic antibody could be sufficient to eradicate human AML LSCs without affecting normal human hematopoiesis.
CD38 [91]	Known to be expressed on most AML and myeloma cells, and its lack of expression on hematopoietic stem cells (HSCs) renders it a potential therapeutic target for relapsed AML.
CD157 [92]	It is a protein expressed in approximately 97% of patients with AML, especially with subtypes M4 and M5 (myelomonocytic and myeloid leukemia). It is considered a valuable marker for minimal residual disease (MRD), and leukemia cells that give rise to relapses have been found to contain CD157. CD157 signaling promotes AML cell survival and may increase resistance to chemotherapy.
CD44v6 [93]	Selectively and highly expressed in hematopoietic and epithelial tumors and is an indication of cancer stem cells for multiple cancers. The CD44 isoform 6 is relatively tumor-limited and associated with a poor prognosis. In AML, CD44v6 was selectively expressed in primary AML cells but not in HSCs, thereby ensuring its safety as a CAR-T therapeutic antigen.
CD47 [94]	It is a protein on leukemia cells (LSCs) and tumor cells that inhibits immune cells (macrophages) from eliminating them. Overexpression of CD47 in AML is linked to increased disease and a worse prognosis. Blocking this signal can overcome this immune suppression, engaging macrophages to eliminate AML cells.
CD64 [96]	Protein surface marker that can be used to identify and treat AML, particularly monocytic forms (AML-M5). The expression of CD64 can be a useful diagnostic tool for distinguishing AML subtypes and is a promising target for new targeted therapies.
U5 snRNP200 [98]	It is a nuclear protein, an essential component of the spliceosome, that acts as an RNA helicase and catalyzes the unwinding of the U4/U6 RNA duplex. It has an abnormal expression on the surface of hematological cancer cells (such as AML) and leukemic B cells. It is not present on normal hematopoietic stem cells.
FLT3 [97]	FLT3 gene mutations occur in 30% of AML cases and are associated with a poorer prognosis, particularly internal tandem duplications (FLT3-ITD).

**Table 3:** Key challenges associated with AML CAR-T therapy.

Challenge	Comments	Advantages	Disadvantages
Target Antigen Selection	Most myeloid antigens (CD33, CD123, CLL-1) are shared with normal hematopoietic stem cells, creating risk of myeloablation	Potential for highly specific targeting. Multiple candidate antigens available.	On-target, off-tumor toxicity Risk of prolonged cytopenias May require stem cell rescue Limited truly leukemia-specific targets
Antigen Heterogeneity and Escape	AML blasts show significant inter- and intra-patient heterogeneity; single-antigen targeting allows antigen-negative relapse	Can use multi-targeted approaches. Combination strategies possible.	Antigen-loss variants emerge Clonal evolution favors escape Requires complex dual/tandem CAR designs
Immunosuppressive Tumor Microenvironment	AML creates hostile bone marrow environment with regulatory T cells, myeloid-derived suppressor cells, and inhibitory cytokines	Can potentially engineer CAR-T cells to resist suppression. Combination with checkpoint inhibitors possible.	CAR-T exhaustion and dysfunction Reduced persistence Impaired trafficking to bone marrow Poor expansion in vivo
Manufacturing Challenges	AML patients often have poor T-cell quality/quantity due to prior chemotherapy and disease burden	Allogeneic CAR-T options in development. Can use healthy donor T cells.	Failed or delayed manufacturing T-cell dysfunction in source material Higher costs and complexity Limited time window in aggressive disease
Disease Kinetics	AML is highly aggressive, with rapid progression compared to B-cell malignancies	Urgent treatment need may accelerate approvals. Clear unmet medical need.	Limited time for CAR-T manufacturing May require bridging chemotherapy Disease progression during production Patients may become too ill for treatment
Cytokine Release Syndrome (CRS)	Risk of severe inflammatory response, though potentially less severe than in ALL due to lower disease burden	Management protocols well-established. Tocilizumab and steroids effective.	Can be life-threatening. Requires ICU-level monitoring May limit dosing Can impact CAR-T efficacy if managed with steroids
Limited CAR-T Persistence	CAR-T cells often show poor long-term persistence in AML compared to lymphoid malignancies	Repeated dosing possible. Can optimize CAR design for persistence.	Higher relapse rates May require consolidation strategies Difficult to maintain remission Unknown optimal dosing schedule
Prior Treatment Effects	Heavy pre-treatment with chemotherapy, hypomethylating agents, and stem cell transplant affects immune function	Multiple lines of therapy validate refractory nature. Supports rationale for novel approaches.	Depleted/exhausted T cell repertoire Impaired CAR-T function Poor manufacturing substrate Increased toxicity risk
Myeloablation Risk	Targeting myeloid antigens risks eliminating normal hematopoiesis, potentially requiring permanent stem cell support	Can plan for stem cell rescue. Haploidentical rescue feasible.	Need for backup stem cells Risk of graft failure Long-term transfusion dependence Complicates risk–benefit analysis
Lack of Reliable Biomarkers	Difficult to predict which patients will respond or develop toxicity	Active area of research. May enable personalized approaches.	Cannot stratify patients effectively Unclear optimal selection criteria Inefficient use of resources Difficult trial design

**Table 4:** Comparative context: AML vs. ALL.

Parameter	B-ALL (Success)	AML (Challenges)
Target antigen	CD19 (lineage-restricted)	CD33, CD123 (myeloid lineage)
TME immunosuppression	Low	High (MDSC, Treg-enriched)
Disease location	Circulating, marrow	Predominantly marrow niches
Metabolic stress	Moderate	Severe (hypoxia, acidosis)
On-target/off-tumor	Manageable (B-cell aplasia)	Severe (myeloablation)

**Table 5:** Strategies to improve AML CAR-T therapy.

Strategy	Description/Comments	Advantages	Disadvantages
Multi-target CAR-T cells	CAR-T cells targeting multiple antigens (e.g., CD33, CD123, CLL-1) simultaneously or sequentially	Reduces risk of antigen escape; broader leukemic cell coverage; improved tumor eradication	Complex manufacturing; potential for increased toxicity; risk of myeloablation requiring stem cell rescue
Logic-gated CAR designs	CAR constructs requiring recognition of multiple antigens (AND gates) or targeting one while avoiding another (NOT gates)	Enhanced specificity; reduced on-target/off-tumor toxicity; protects normal hematopoietic cells	Complex engineering; potentially reduced CAR-T activation; technically challenging to manufacture
Switchable/controllable CARs	Systems using adapter molecules or small molecule switches to control CAR-T activity	Titratable activity: can be turned off if toxicity occurs; improved safety profile	Requires continuous administration of adapter molecules; increased complexity and cost; potential immunogenicity
Armored CAR-T cells	CAR-T cells engineered to secrete cytokines (IL-15, IL-18) or express chemokine receptors	Enhanced persistence and proliferation; improved trafficking to tumor sites; resistance to immunosuppressive microenvironment	Risk of cytokine-related toxicity; potential for uncontrolled T-cell expansion; manufacturing complexity
Allogeneic (off-the-shelf) CAR-T	Universal donor-derived CAR-T cells with edited TCR and HLA to prevent GVHD and rejection	Immediate availability; reduced cost; standardized product; no need for patient leukapheresis	Risk of rejection; limited persistence; GVHD potential; requires sophisticated gene editing
Combination with chemotherapy	CAR-T therapy combined with lymphodepleting or targeted chemotherapy	Reduces tumor burden pre-infusion; lymphodepletion enhances CAR-T expansion; synergistic effects	Added toxicity; complexity in timing and dosing; may affect CAR-T cell quality if given before collection
Checkpoint inhibitor combination	CAR-T cells combined with PD-1/PD-L1 or CTLA-4 inhibitors	Prevents T-cell exhaustion; enhances CAR-T persistence; overcomes immunosuppressive microenvironment	Increased immune-related adverse events; potential for severe toxicity; cost implications
Targeting AML stem cells	CARs directed against stem cell markers (CD33, CD123, TIM-3, CD96)	Addresses root cause of relapse; potential for curative outcomes; prevents disease regeneration	Risk of hematopoietic stem cell depletion; requires stem cell transplant backup; long-term cytopenias
NK cell-based CAR therapy	CAR-engineered natural killer cells instead of T cells	Lower cytokine release syndrome risk; can use allogeneic sources; natural antitumor activity	Limited persistence compared to T cells; less clinical experience; expansion challenges
Suicide gene integration	Incorporation of safety switches (e.g., inducible caspase-9) for rapid CAR-T elimination	Enhanced safety; ability to quickly terminate therapy if severe toxicity occurs; patient reassurance	Irreversible elimination of CAR-T cells; may lose therapeutic benefit; requires additional genetic modification
Autologous stem cell backup	Collection and preservation of patient’s stem cells before CAR-T therapy	Allows aggressive targeting of myeloid antigens; safety net for myeloablation; enables hematopoietic rescue	Requires additional procedures; increased cost; stem cells may be contaminated with leukemic cells
Base editing/CRISPR enhancement	Precise gene editing to enhance CAR-T function, reduce exhaustion, or improve specificity	Highly specific modifications; can knockout inhibitory receptors; improved CAR-T functionality	Technical complexity; potential off-target effects; regulatory challenges; long-term safety unknown
CAR-NK or CAR-macrophage therapy	Using innate immune cells as CAR platforms instead of T cells	Different toxicity profile; can target tumor microenvironment; complementary mechanisms	Less established clinical data; persistence questions; manufacturing challenges

## Data Availability

Not applicable.
